# Oligosaccharides Derived from Red Seaweed: Production, Properties, and Potential Health and Cosmetic Applications

**DOI:** 10.3390/molecules23102451

**Published:** 2018-09-25

**Authors:** Kit-Leong Cheong, Hua-Mai Qiu, Hong Du, Yang Liu, Bilal Muhammad Khan

**Affiliations:** Guangdong Provincial Key Laboratory of Marine Biotechnology, STU-UNIVPM Joint Algal Research Center, Department of Biology, College of Science, Shantou University, Shantou 515063, China; klcheong@stu.edu.cn (K.-L.C.); 18hmqiu@stu.edu.cn (H.-M.Q.); hdu@stu.edu.cn (H.D.); liuyanglft@stu.edu.cn (Y.L.)

**Keywords:** red seaweed, agaro-oligosaccharides, carrageenan-oligosaccharides, biological activities

## Abstract

Because of their potential use as functional ingredients in human nutrition, oligosaccharides derived from natural sources are receiving paramount consideration. Red seaweed, a proven rich source of agar and carrageenan, is one of the most abundantly present sources of such oligosaccharides. Agaro-oligosaccharides (AOS) and carrageenan-oligosaccharides (COS) are produced from agar and carrageenan, respectively, through chemical and enzymatic hydrolyses. Enzymatic hydrolysis of agar and carrageenan into oligosaccharides is preferred in industrial production because of certain problems associated with chemical hydrolysis, including the release of high amounts of monosaccharides and undesirable toxic products, such as furfural. AOS and COS possess many biological activities, including prebiotic, immuno-modulatory, anti-oxidant, and anti-tumor activities. These activities are related to their chemical structure, molecular weight, degree of polymerization, and the flexibility of the glycosidic linkages. Therefore, the structure–function relationship and the mechanisms occurring during the specific biological applications of AOS and COS are discussed herein. Moreover, the chromatographic separation, purification, and characterization of AOS and COS are also part of this review. This piece of writing strives to create a new perspective on the potential applications of AOS and COS in the functional food and pharmaceutical industry.

## 1. Introduction

Phylogenetically, red seaweed is the oldest division of marine macrophytes. It is found in abundance in oceans and has gained much attention as an important marine vegetation since the late 1990s. Polysaccharides extracted from red seaweed differ in their composition and structure from all other polysaccharides of plant origin, including those obtained from other marine algae [1]. Their polysaccharides contain unique galactans, normally divided into two groups: Agar and carrageenan [2,3]. The backbone of these polysaccharides is a repeating disaccharide unit of 3-linked β-d-galactopyranose and 4-linked α-galactopyranose residues. Carrageenan belongs to d-series in α-galactose residues, whereas agar belongs to the l-series [4]. A substantial part or even all of the α-galactose residues may exist in the form of 3,6-anhydro-galactose (AHG). The structure of carrageenan varies in degrees of sulfation and is divided into three major forms, including κ-, ι-, and λ-carrageenan [5]. κ-Carrageenan is composed of repeating disaccharide units of 3-linked 4-sulfate-β-d-galactopyranose (G4S) and 4-linked AHG. ι-Carrageenan and λ-carrageenan have a similar structure to κ-carrageenan, but with two and three sulfate groups, respectively.

**Figure 1:**
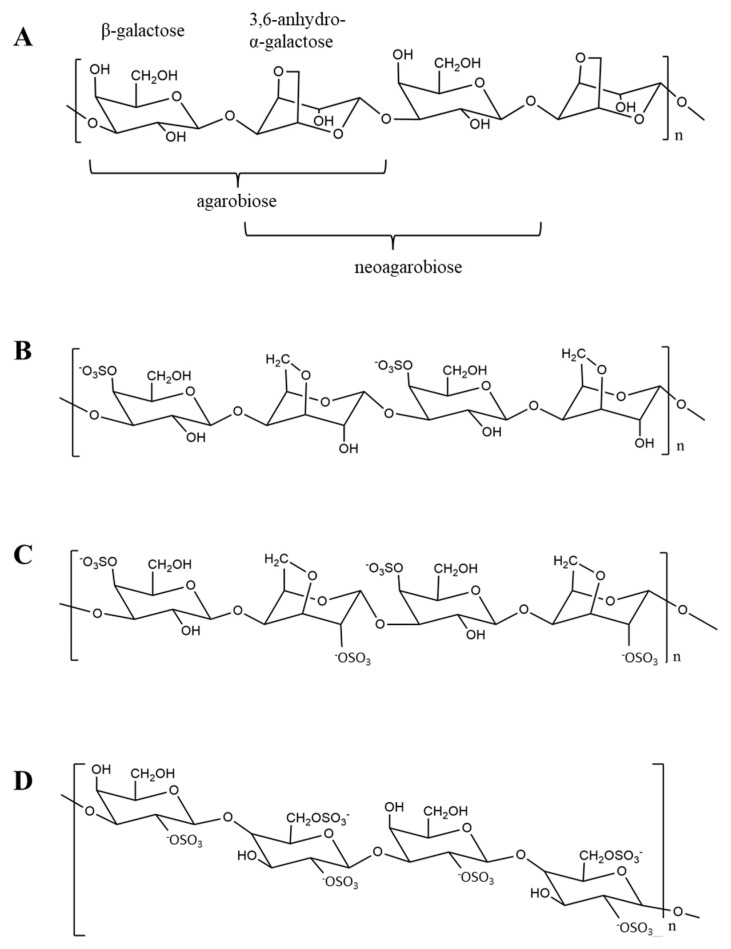
Schematic structure of red seaweed oligosaccharides. (**A**) Agaro-oligosaccharide and neoagaro-oligosaccharides; (**B**) κ-carrageenan oligosaccharides; (**C**) ι-carrageenan oligosaccharides; and (**D**) λ-carrageenan oligosaccharides.

Chemical or enzymatic hydrolysis of agar and carrageenan yield red algae oligosaccharides [6,7]. Agar oligosaccharides are classified into agaro-oligosaccharides (AOS) and neoagaro-oligosaccharides (NAOS). The non-reducing terminal end of AOS is a galactose unit, whereas that of NAOS is an AHG unit [8] (Figure 1). On the other hand, carrageenan oligosaccharides (COS) are classified into κ-, ι-, and λ-carrageenan oligosaccharides according to the number and position of the sulfate groups [9] (Figure 1). Due to the tremendous potential of these oligosaccharides with respect to various biological activities, the number of scientific literature reporting such bioactivities is continuously growing since 2000. AOS and COS are considered non-digestible oligosaccharides, which are non-cariogenic in humans, and are reported to possess prebiotic, anti-tumor, anti-oxidant, and immuno-modulatory properties [10,11]. Usually, the bioactivities of oligosaccharides are closely correlated with their molecular properties, such as the degree of polymerization (DP), molecular size, type and ratio of constituent monosaccharides, and anomeric configuration and position of glycosidic linkages [12,13]. A basic understanding of the chemico-physical properties and biological activities of AOS and COS is essential for their successful application in functional foods and other multifunctional applications.

Many other varieties of oligosaccharides, such as fructo-oligosaccharides, xylo-oligosaccharides, manno-oligosaccharides, and galacto-oligosaccharides, are widely used in food, pharmaceutical products, and animal feed, and as drug carriers [14,15,16,17]. However, few reviews have focused on red seaweed oligosaccharides, such as AOS and COS, even though these oligosaccharides possess superior biological activities, and despite the high yield of seaweed [18]. Here, we present an overview of this topic to review advances in this field, with an emphasis on the production, purification, analysis, characterization, and biological properties of AOS and COS.

## 2. Production of Oligosaccharide from Red Seaweed

### 2.1. Pretreatment of Red Seaweed Polysaccharides

**Figure 2:**
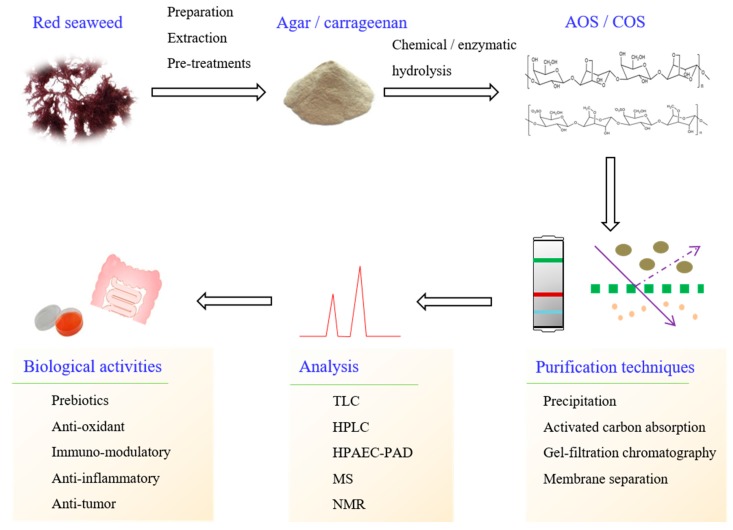
Schematic diagram of red seaweed oligosaccharides production, purification, analysis, and their resultant biological activities.

The extraction of polysaccharides, however, is preceded by a number of pretreatments, so that the polysaccharides are obtained in their pure form (Figure 2). To eliminate impurities, such as low molecular weight compounds, colored matter, and lipids, powdered red seaweed is subjected to washing with ethanol or chloroform before extraction. The methods for polysaccharide extraction from red seaweed include thermal, microwave-assisted, ultrasonic-assisted, and enzymatic assisted extraction [19,20,21,22]. To obtain high amounts of polysaccharide from red seaweed, high efficiency extraction methods, such as ultrasonic-assisted and microwave-assisted extraction, have received a great deal of attention as potential alternatives to conventional hydrothermal methods. These two extraction methods have shown a higher efficiency and shorter extraction time. The red seaweed polysaccharides are afterwards obtained through ethanol precipitation.

### 2.2. Chemical and Enzymatic Hydrolyses for Oligosaccharides Production

Since naturally occurring AOS and COS are scarce in number, in vitro de-polymerization of red seaweed polysaccharides is often resorted to as a reliable strategy for producing oligosaccharides. Chemical- and enzyme-assisted hydrolysis of red seaweed polysaccharides are the two main methods for AOS and COS production, each with its own distinctive advantages and disadvantages. The former is considered useful because it is affordable, simple, and fast. However, chemical hydrolysis is non-specific, meaning it can produce high amounts of monosaccharides with the accompanying production of the undesirable toxic compound, furfural [15]. Enzymatic hydrolysis, on the other hand, potentially offers many advantages, such as specific hydrolysis resulting in the production of high amounts of target oligosaccharides, is environmentally friendly, and produces low amounts of monosaccharides and toxic molecules. The obvious disadvantage is the low activity of the enzyme that requires a long reaction time. Other disadvantages include the high price of the enzyme, difficulty associated with its reuse, and spontaneous loss of enzymatic activity.

#### 2.2.1. Oligosaccharides Production by Chemical Hydrolysis and De-Polymerization

A chemical method using diluted acids, such as hydrochloric acid, nitric acid, sulfuric acid, trifluoroacetic acid, formic acid, tartaric acid, and lactic acid, after being heated to 50–90 °C, can be employed for the production of oligosaccharides from red seaweed polysaccharides, such as agar [23,24] and carrageenan [6,25]. A random cleavage of glycosidic linkage is observed after de-polymerization with an acid solution. An acid solution of 1 mol·L^−1^ TFA has been used to partially hydrolyze commercial agar and carrageenan at 65 °C for 3 h to obtain AOS and COS [26]. Similarly, a series of AOS have been obtained from agar through hydrolysis with 0.1–0.8 mol·L^−1^ HCl at 50 °C for 6 h [24]. Likewise, COS have been obtained by mild acid hydrolysis (0.1 mol·L^−1^ TFA at 37 °C for 24 h) of κ-carrageenan from *Chondrus armatus*, and the resultant oligosaccharides showed anti-inflammatory activity [27].

In addition to acid hydrolysis, hydrogen peroxide can also be used for de-polymerization of red seaweed polysaccharides. In fact, a number of low molecular weight oligosaccharides from carrageenan, with different structural types, have been obtained using H_2_O_2_ de-polymerization at 37 °C [28]. The structures and biological activities of oligosaccharides obtained with H_2_O_2_ and HCl hydrolysis, though, are quite different. For instance, the structure of COS obtained through H_2_O_2_ hydrolysis is usually –(G4S–AHG)_n_–, –AHG–(G4S–AHG)_n_–, –(G4S–AHG–OOH)_n_–, and –AHG– (G4S–AHG–OOH)_n_–, whereas that of the same oligosaccharides obtained through HCl hydrolysis is frequently –G4S–(AHG–G4S)_n_– and occasionally –(G4S–AHG)_n_–. Moreover, the H_2_O_2_ hydrolysates have been found to exhibit much higher antioxidant activities than the HCl hydrolysates [6]. Kim et al. reported that the maximum reducing sugar yield (7.47 g/L, 37.49%) was obtained from *Gracilaria verrucosa* with 0.1 mol·L^−1^ HCl at 121 °C for 15 min, while under a larger-scale hydrolysis process a yield of 10.63 g/L (21.26%) was obtained for reducing sugar [29].

Ultrasonic-assisted and microwave-assisted hydrolysis have received considerable attention as potential alternatives to conventional hydrothermal methods due to their inherent advantages, including higher efficiency, shorter extraction time, reduced solvent requirements, and lower cost [30,31]. Microwave-assisted HCl hydrolysis at 100 °C for 15 min has been successfully employed for the production of COS [32]. The chemical hydrolysis of polysaccharides is regarded as the best choice for oligosaccharide production on an industrial scale due to it is simplicity, affordability, and reproducibility. However, the disadvantages inherent in its use are its lack of specificity and the associated environmental hazards.

#### 2.2.2. Oligosaccharides Production by Enzymatic Hydrolysis

Enzymatic hydrolysis to produce AOS and COS is an alternative to chemical hydrolysis for the same purpose. Although this method requires special equipment, it does not form undesirable toxic by-products or high amounts of monosaccharides as observed in chemical hydrolysis. The enzymes used for specific hydrolysis to produce AOS and COS from red seaweed polysaccharide include α-agarase (EC 3.2.1.158) [33], β-agarase (EC 3.2.1.81) [24], β-porphyranase (EC 3.2.1.178) [34], β-galactosidase (EC 3.2.1.23) [35], κ-carrageenase (EC 3.2.1.83) [36], ι-carrageenase (EC 3.2.1.157) [37], λ-carrageenase (EC 3.2.1.162) [38], cellulase (EC 3.2.1.4) [39], and pectinase (EC 3.2.1.15) [40]. These enzymes are produced by microorganisms from many sources, including seawater, marine algae, marine mollusks, soil, and the human gut. Microorganisms living on marine algae use polysaccharides as a carbon source for the production of energy. Therefore, the microorganisms that produce certain enzymes for the hydrolysis of red seaweed polysaccharides are mainly found in marine environments. Only a few agarolytic bacteria can be isolated from terrestrial environments. The methods for isolating these microorganisms, purifying the enzymes, and measuring the enzymatic activity are summarized in previous reports [8].

Different glycosidases exhibit different mechanisms for the hydrolysis of red seaweed polysaccharides. For instance, α-agarase hydrolyzes α-1,3 linkages and produces AOS with AHG at the reducing end. Conversely, β-agarase digests β-1,4 linkages and produces NAOS with β-d-galactose at the reducing end [7,8]. Furthermore, β-porphyranases hydrolyze the β-(1,4)-glycosidic bonds of the porphyran repetition moieties of galactose linked with G4S in agar and produce oligosaccharides with galactose residues at the reducing ends [41]. Carrageenases, on the other hand, are highly specific for hydrolyzing carrageenans to produce COS. For example, κ-, ι-, or λ-carrageenase only degrades κ-, ι-, or λ-carrageenan, respectively [36,42]. These enzymes specifically endo-hydrolyze the β-(1,4)-glycosidic linkage between AHG and galactose, producing a series of COS. Vanegas et al. reported a reducing sugar yield of 18 g/L for *Laminaria digitata* under the enzymatic hydrolysis process at 37 °C for 24 h [43]. The enzymatic hydrolysis process produced a high amount of oligosaccharide but the reaction time required is considerably longer.

Many reports have described the production of AOS and COS using the enzymatic method. Recombinant β-agarase (Aga 50A) from *Escherichia coli* hydrolyzes the *Gracilaria* spp. agar to produce higher amounts of AOS than commercial agarase [44]. Cui et al. isolated a novel β-agarase from the marine bacterium, *Catenovulum agarivorans* YM01T, that could hydrolyze the β-1,4-glycosidic linkages of agar, and obtained neoagarotetraose and neoagarohexaose as the main products. This β-agarase was stable below 50 °C and retained 13% of the original activity after being heated to 80 °C for 1 h [45]. β-Galactosidase from *Vibrio* sp. EJY3 has been found to produce odd-numbered AOS from agar [35]. Likewise, agar has been reportedly digested by Celluclast 1.5-L (cellulase from *Trichoderma reesei*) and agarase to produce AOS and NAOS, respectively, which exhibited 2,2-diphenyl-1-picrylhydrazyl (DPPH) and 2,2′-azino-bis-(3-ethylbenzothiazoline-6-sulphonic acid) (ABTS) radical scavenging activities, which was lower than the ascorbic acid as the negative control [46,47].

The immobilized enzyme technique has been used in industrial applications to obtain oligosaccharides in batches in a continuous process. The advantages of immobilized enzymes include the ease of enzyme recovery from the reaction mixture, ease of controlling the de-polymerization product, rapid termination of the enzyme reaction, and feasibility for application in various engineering designs [48]. For instance, an immobilized agarase, obtained from *Pseudomonas aeruginosa* AG LSL-11, appeared to be more stable and had higher activity compared to the free enzyme [49]. Similarly, Xiao et al. reported a ҡ-carrageenase that was immobilized onto magnetic iron oxide nanoparticles. The thermal, pH, and storage stability of this immobilized ҡ-carrageenase was relatively higher than that of free ҡ-carrageenase [50].

Enzymatic hydrolysis is seen as the best option for producing AOS and COS for the food, pharmaceutical, and cosmetic industries. Therefore, demand for hydrolytic enzymes is growing rapidly, and this demand is driving research on enzymes that are inexpensive, highly specific, and that have high activity levels [51,52].

## 3. Purification of Oligosaccharides

The production of AOS and COS by either of the aforementioned methods may be accompanied by some impurities or unwanted compounds, such as monosaccharides, protein, acid, and furfural. The production of food-grade or pharmaceutical-grade oligosaccharides necessitates purification of the reaction mixture to obtain the highest possible purity for the resultant AOS or COS. The desired purity of oligosaccharides depends on the different uses of oligosaccharides in various fields.

**Figure 3:**
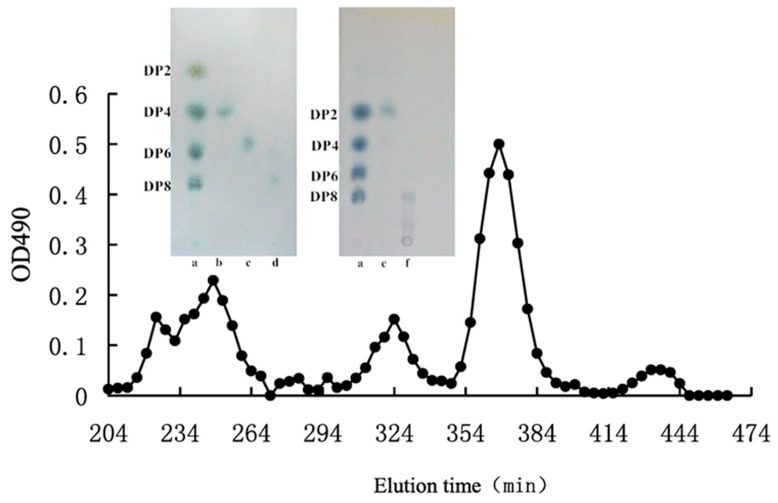
The purification of neoagaro-oligosaccharides by Sephadex LH-20 chromatography (100 × 1.6 cm). Distilled water was the elution phase and the fraction was detected by phenol-sulfuric acid method. The purity of the purified neoagaro-oligosaccharides (NAOS) was proved by thin layer chromatography (TLC) assay (inserted panel). The figure is from reference [23].

The AOS and COS purification procedures include ethanol precipitation [53], activated carbon adsorption [54], membrane separation [24], and gel filtration chromatography [11]. The enzyme in the reaction mixture is precipitated by low concentrations of ethanol, whereas salts and other small molecules are removed using the membrane separation method [55]. The oligosaccharides, however, are insufficiently pure after the ethanol precipitation, activated carbon adsorption, and membrane separation procedures. Pure oligosaccharides with different molecular weights, nonetheless, are obtained by gel filtration chromatography. In fact, a series of AOS, digested from *Porphyra umbilicalis* polysaccharides, have been purified through three Superdex 30 (26/60) gel filtration columns in series [56]. Other size-exclusion columns, including Superdex peptide 10/300 GL [57], Toyopearl HW-40C [58], Bio-gel P-6 [28], Bio-gel P-2 [59], and Sephadex LH-20 [23], have also been successfully used to produce very pure AOS and COS. An illustration of oligosaccharide purification by size-exclusion chromatography is provided in Figure 3.

The purity of oligosaccharides is one of the important quality indices for the potentiation of their biological effects, and for the production of a commercial product. Gel filtration chromatography produces high purity oligosaccharides with the same degree of polymerization. However, the disadvantages of this approach are that it is expensive and is difficult to reproduce on a large-scale. Membrane separation and precipitation can generate oligosaccharide products on a large-scale, but the purity of the oligosaccharides is inferior as these methods only obtain mixtures of oligosaccharides with diverse molecular weights.

## 4. Chromatographic Separation of Oligosaccharides

Several chromatographic methods, such as thin layer chromatography (TLC), high-performance liquid chromatography (HPLC), gas chromatography (GC), high-performance anion exchange chromatography-pulsed amperometric detector (HPAEC-PAD), and capillary electrophoresis (CE), have been used for the separation of AOS. TLC offers some advantages due to its relatively simple sample preparation and because it is comparatively inexpensive for separating AOS on a silica G60 plate [11]. For instance, a silica G60 plate was developed using a solvent system of butanol, ethanol, and water in a ratio of 2:1:1, which was then visualized by spraying the plates with a staining solution of 1% diphenylamine and aniline in acetone [60]. Due to the low resolution of TLC and the difficulty associated with proper quantification of AOS, TLC is only suitable for preliminary analyses.

The most commonly employed method for the separation of AOS is HPLC due to its precision and consistency [61,62]. Because of the inability of AOS to absorb ultraviolet (UV) radiation, a refractive index detector (RID) [63,64] and evaporative light scattering detection (ELSD) [65] are usually used during HPLC analyses. Although RIDs are recommended as simple and universal detectors, these detectors, nonetheless, suffer from several drawbacks. RIDs are not compatible with gradient elution, and the mobile phase requires a long time to achieve baseline stabilization. Hydrophilic interaction chromatography (HILIC) in HPLC involves the use of hydrophilic stationary phases and a hydrophobic mobile phase. The stationary phase is a material of silica or polymer particles modified with different types of polar functional groups, such as amino, amide, diol, cyano etc., while the mobile phase is in combination with the organic mobile phase (usually acetonitrile) and elution is performed by increasing the water concentration [66,67]. For example, the AOS, produced from agar hydrolyzed by immobilized agarase, can be separated by an amino column using 0.1% formic acid in acetonitrile as the mobile phase [49].

High-performance size-exclusion chromatography (SEC) can also be used for separation of AOS. In fact, the AOS hydrolyzed from porphyran have been separated by a TSK G3000PWxl size-exclusion column and detected by a refractive index detector (RID) wherein the mobile phase for isocratic elution consisted of 0.2 mol·L^−1^ sodium sulfate aqueous solution [68]. Similarly, a peptide 10/300 GL column with an ammonium bicarbonate mobile phase has been successfully used to separate AOS with a DP of two to eight, which were hydrolyzed by β-agarase [60]. Likewise, a Supelco Gel 610-H column with 0.1% (*v*/*v*) aqueous phosphoric acid as the mobile phase has also been employed for the separation of AOS produced from agarose wherein the HPLC chromatogram identified three oligosaccharides with molecular weights of 77,791, 2,311, and 328 Da [63]. Moreover, Kim et al. used a Shodex KS-802 column for neo-agarobiose analysis, which was hydrolyzed using exo-agarase [69].

In addition, HPLC coupled with a diode array detector (DAD) or fluorescence detector, having the ability to identify compounds with an absorption range of 190 to 380 nm, can also be used for the separation of oligosaccharides. For instance, AOS and COS have been fluorescence-labeled with 2-aminobenzamide and detected by a fluorescent detector [60], and derivatized with α-naphthylamine and detected by a DAD detector [70]. Likewise, reversed phase column has been successfully used to separate derivatized AOS and COS wherein AOS, ranging from two to eight DP, were separated using a BEH C18 column with water, ethanol, and 20 mmol·L^−1^ heptylammonium as the mobile phase [34].

Correspondingly, for CE analysis, oligosaccharides should first be converted into UV-absorbing chromophores or fluorescent derivatives [71]. Despite CE enabling high-resolution separation of heterogeneous mixtures of oligosaccharides [72], only a few works report CE analysis of AOS. The recent developments in CE techniques for carbohydrate analysis has previously been reviewed [73]. Polysaccharide analysis using carbohydrate gel electrophoresis (PACE) is a common separation technique used to separate oligosaccharides from natural products [74]. PACE relies on the derivatization of oligosaccharides with a fluorophore, allowing separation through gel electrophoresis and detection through florescence imaging of the polyacrylamide gel. The reagent used for derivatization is often 8-aminonapthalene-1,3,6-trisulfonic acid. For instance, the separation of low molecular weight COS, with DP of 2 to 17, hydrolyzed from red seaweed carrageenan has been successfully achieved using PACE [75]. The obvious advantages of florescence labelling of oligosaccharides are that it provides an excellent resolution and sensitivity as well as highly reproducible quantification [76].

The HPAEC-PAD approach is highly sensitive and offers good resolution for the detection of oligosaccharides [77]. Kazłowski et al. have investigated NAOS and AOS mixtures with a DP of 2 to 24 by using HPEAC-PAD [24]. Likewise, NAOS with a DP of 2 to 20 have been separated on a carbopac PA100 (4 × 250 mm) column, using 150 mmol·L^−1^ NaOH as the mobile phase, and detected by an electrochemical detector with a gold electrode [78]. However, HPAEC-PAD might not be suitable for routine analysis because the technique requires a specific instrument, the detector performance depends on the condition of the PAD electrode with the response decreasing as a function of the number of injections, and the process is labor-intensive [79].

## 5. Physicochemical Characteristics

Mass spectrometry (MS) is a sensitive and powerful detection tool for elucidating the oligosaccharide structure. The detailed oligosaccharide information that can be obtained from MS includes an accurate molecular weight, chain length distribution, fragments information, monosaccharide compositions, linkages, and location of various modifications [9,80]. Electrospray ionization (ESI) and matrix-assisted laser desorption/ionization (MALDI) have advanced the structural analysis of AOS and COS, as these two ionization techniques are suitable for examining low and middle molecular weight oligosaccharides. AOS and COS include repeating 3-linked β-d-galactopyranose and 4-linked α-galactopyranose residue units, but COS are different in the sulfation substitute, therefore, their fragmentation patterns are different in ESI tandem MS [9].

Fragmentation patterns for the three different types of COS, including κ-, ι-, and λ-COS, can be determined using negative ion collision-induced dissociation tandem MS. Using this method, it has been determined that the disaccharide units of the three types of COS have different and unique masses: 386 Da for κ-COS (G4S-AHG), 466 Da for ι-COS (G4S-AHG-S), and 564 Da for λ-COS (G2S-D2S6S) [81]. Similarly, the isomeric disaccharide differentiation of COS has been achieved using collision-induced dissociation tandem MS for [M-Na]^−^ and for B_1_ ions [81].

**Figure 4:**
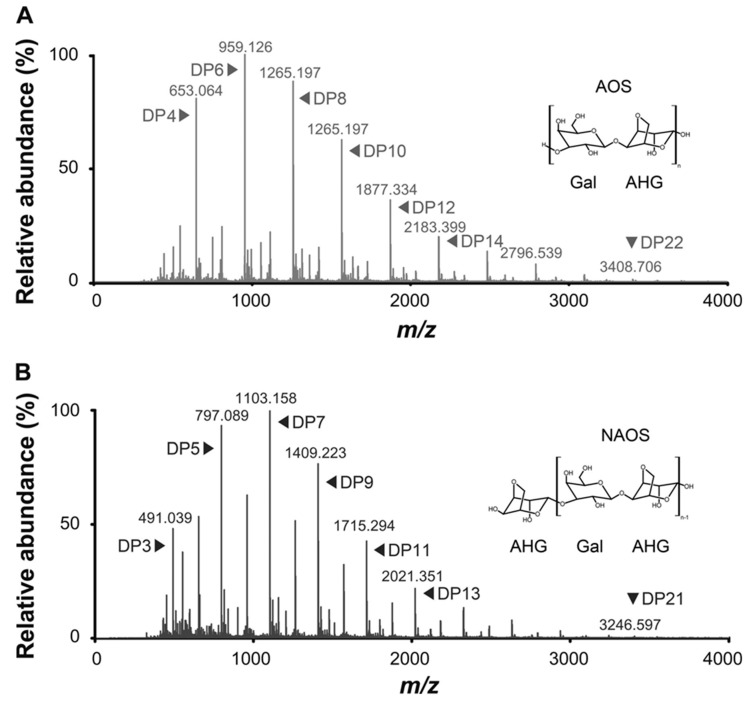
Representative MALDI-TOF/TOF MS of agaro-oligosaccharide (**A**) and neoagaro-oligosaccharide (**B**) prepared by acid hydrolysis of agarose. Figure is from reference [82].

Compared with ESI-MS, MALDI-MS produces mostly singly-charged ions for the analysis of oligosaccharides. The measurements are usually performed by adding the matrix onto the dried sample spot. The use of a matrix, such as 2,5-dihydroxybenzoic acid (DHB), 3-aminoquinoline (3-AQ), or 2,4,6-trihydroxyacetophenone (THAP), may favor the more hydrophobically-labeled samples. A MALDI-TOF/TOF MS analysis for a series of even-numbered AOS produced by acid prehydrolysis of agar has been performed using a DHB matrix wherein the hydrolysates were characterized as agarotetraose, agarohexaose, and agarooctaose [82]. The representative spectra of MALDI-TOF/TOF MS have been shown in Figure 4. It has been established from MALDI-TOF mass spectra that neo-agarotetraose, neo-agarohexaose, and neo-agarooctaose have molecular ions at a *m*/*z* of 653 [M + Na]^+^, 959 [M + Na]^+^, and 1265 [M + Na]^+^, respectively [83].

**Figure 5:**
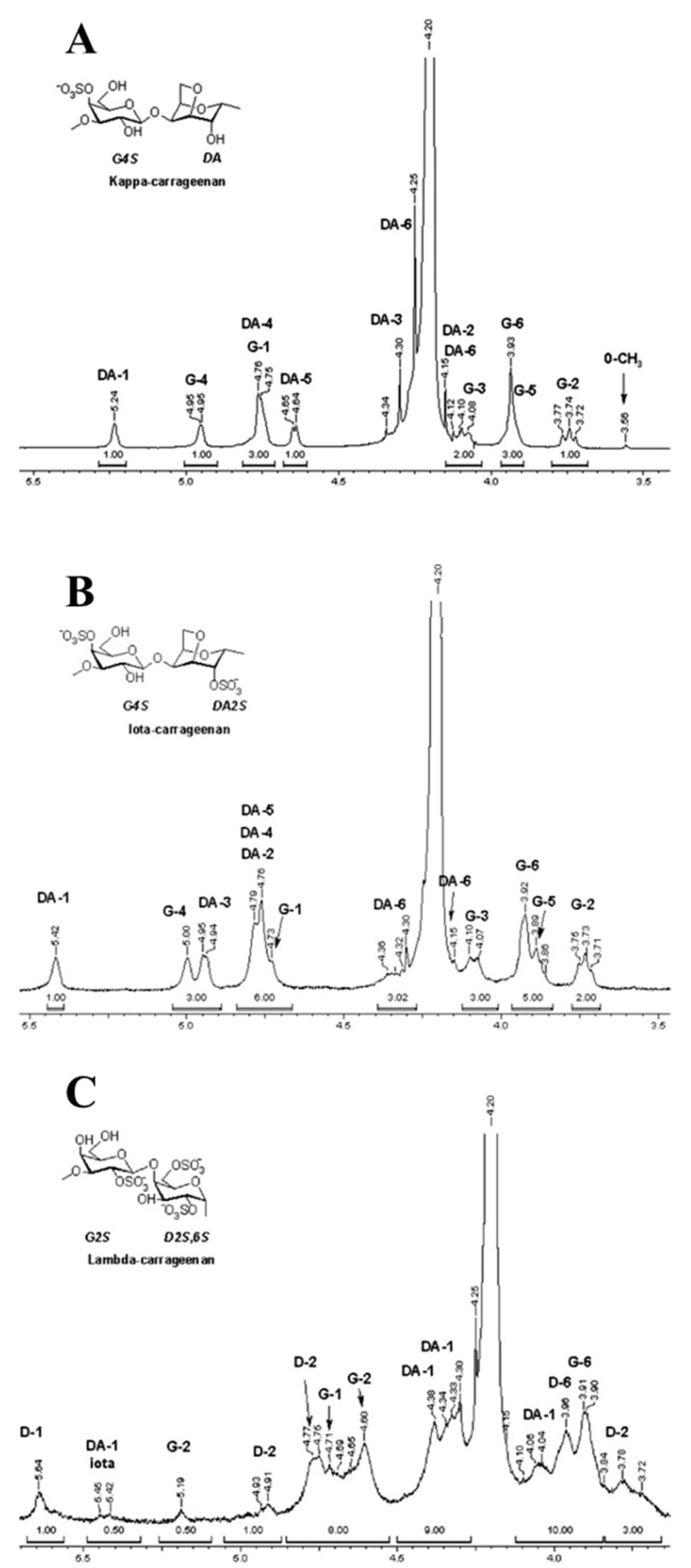
Represent ^1^H-NMR of (**A**) κ-carrageenan, (**B**) ι-carrageenan, and (**C**) λ-carrageenan in deuterium oxide. D: 4-linked α-galactopyranose; DA: 4-linked 3,6-anhydro-α-galactose; G: 3-linked β-d-galactopyranose. The figure is from reference [86].

Nuclear magnetic resonance (NMR) spectroscopy is a powerful technique for structurally analyzing oligosaccharides. NMR data provide information about the oligosaccharide structure, including the monosaccharide composition, the presence of α- or β-type carbohydrates, linkage features, and the sequence of the monosaccharide units [84]. For 1D NMR (^1^H-NMR and ^13^C-NMR) spectrum, a signal in the region of 5.1–5.7 ppm is assigned to the α-configuration, whereas the β-configuration appears in the range of 4.5–4.8 ppm in ^1^H-NMR spectrum. For example as shown in Figure 5, the 3-linked β-d-galactopyranose is in the range of 4.7–4.8 ppm, while the 4-linked α-galactopyranose and 4-linked 3,6-anhydro-α-galactose are in the range of 5.2–5.6.

Furthermore, two-dimensional (2D) NMR, such as H-H correlation spectroscopy (COSY), has been used to determine the chemical shift of each proton and its accurate position in the spectra. C–H correlation methods, including spectrum heteronuclear single-quantum correlation spectroscopy (HSQC) and heteronuclear multiple-bond correlation spectroscopy (HMBC), are used to analyze the linkage positions of carbohydrates and the linking relationships with each other. The proton and carbon assignments for a series of COS, produced with carrageenases isolated from the marine bacteria, *Alteromonas fortis* and *Pseudolalteromonas atlantica*, have been obtained from one-dimensional (1D) NMR (^1^H- and ^13^C-NMR) and 2D NMR, including COSY, HMQC, and HMBC [85]. Likewise, the corresponding carbons of a series of AOS, degraded with β-porphyranase A from a marine flavobacterium, have been deduced from HMQC, and the connections between H4 and H5–H6 systems have been obtained using HMBC. The results proved the AOS had a L6S-G disaccharide structure [56].

## 6. Biological Activities of Oligosaccharides

Numerous studies have revealed the inherent beneficial effects of AOS and COS for human health, including prebiotic effects [10,11], immuno-modulatory [87], anti-inflammation [88], anti-oxidant [6,46], and anti-tumor [89] activities. A compilation of some noteworthy observations in this direction is presented in the following paragraphs.

**Figure 6:**
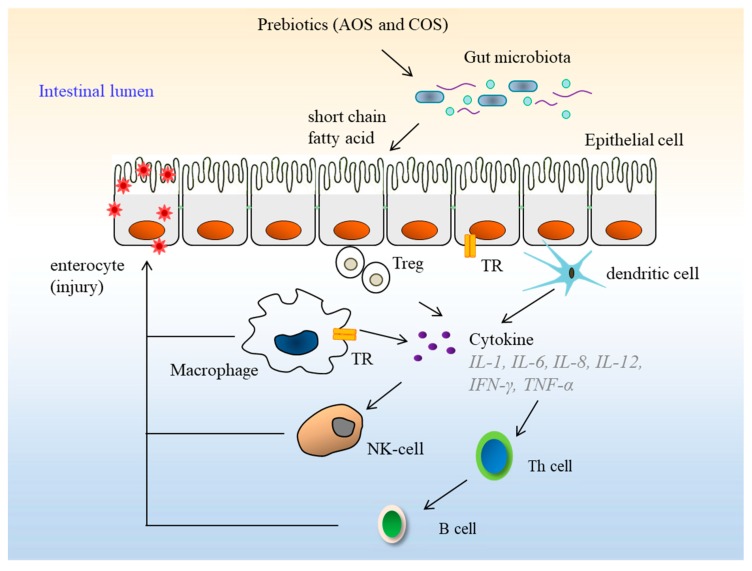
Schematic representation of agaro-oligosaccharide and carrageenan-oligosaccharide as prebiotics and their activity in host immuno-modulation. IL: Interleukin; TNF-α: Tumor necrosis factor-α; TR: Toll-like receptor.

The human gut is one of the most diverse bacterial ecosystems. These bacteria live in a mutually beneficial association with the host. Prebiotics are non-digestible ingredients that beneficially affect host health by selectively stimulating the growth and activity of one or a limited number of beneficial bacteria in the colon [90]. AOS have been reported to possess prebiotic effects. For instance, the incidence of high-fat-diet-induced gut dysbiosis can be reduced or even prevented by administering a mixture of prebiotic AOS, which reportedly averts a change in the microbial composition induced by consumption of a high-fat diet [89]. Similarly, *Bacteroides uniformis* L8, isolated from human feces, has shown a significant activity in digesting AOS and generating galactose as the end product. *B. uniformis* plays a significant role in mitigating high-fat-diet-induced metabolic disorders. AOS derived from agar have shown potential prebiotic effects and may be suitable for therapy targeting obesity-related metabolic disorders [11]. Additionally, COS have also been reported to be degraded by *Bacteroides* isolated from human gut microbiota [91]. Human colonic microflora can interrelate with oligosaccharide to generate bioactive metabolites, in which the microbial metabolites of oligosaccharides, especially short chain fatty acids, have great influence on host physiology and energy homeostasis [92]. Prebiotics and beneficial bacteria are capable of inhibiting pathogens and regulating the host immune system. Prebiotics are processed and presented into the immune system where they modulate the innate and adaptive response (Figure 6). AOS and COS have a range of immune-modulatory and anti-inflammatory effects in mucosal and metabolic tissues. COS can protect microglial cells from being activated by lipopolysaccharide (LPS), and its inhibitory function is related to the sulfate group content of COS [87]. Likewise, carrageenan and COS have been reported to stimulate the induction of interleukin (IL)-10 in human and mice blood cells, with COS inducing more IL-10 in vivo than carrageenan [93]. NAOS with a DP of four could significantly reduce the production of proinflammatory cytokines, such as TNF-α and IL-6, and release nitric oxide (NO) in LPS-induced macrophages. This oligosaccharide may reduce the inflammatory responses by down-regulating the mitogen-activated protein kinase (MAPK) and NF-κB signaling pathways in LPS-stimulated macrophages [88]. A pentasaccharide, de-polymerized from κ-carrageenan, has been shown to significantly reduce Aβ25-35-induced loss of cell viability and apoptosis. This COS, with a DP of four, can down-regulate the protein expression levels of Aβ25-35-induced cleavage caspase 3 by inhibiting the over-activation of the Jun amino-terminal kinases (JNK) signaling pathway [94].

Oligosaccharide samples are able to scavenge different radicals, such as DPPH and ABTS radicals, in vitro. The hydroxyl groups in positions C-2 and C-6 in oligosaccharides are mainly involved in H-atom transfer reactions with these radicals [95]. A series of AOS, degraded from agar by cellulose, have exhibited considerable radical scavenging activities for DPPH and ABTS. A higher degree of hydrolysis could increase the anti-oxidant activities [46,47]. COS obtained by degradation of parent ҡ-carrageenan have demonstrated anti-oxidant activities, which could be related to the DP, the content of reducing sugar, sulfate groups, and the reducing terminus of COS [6].

## 7. Potential Use of Oligosaccharides in Cosmetics

Since time immemorial, human society has relied upon the use of cosmetics for religious as well as ornamental purposes. Earlier civilizations extracted such products from natural compounds (milk, flowers, fruits, seeds, vegetables, etc.) and minerals (clay, ash, etc.) [96]. The growing understanding of the phenomena behind skin damage and aging, and the utilization of natural products against such damages highlighted the importance of biocosmetics as skin-protectives rather than mere ornamentals.

The direct exposure of skin to the environment makes it sensitive and vulnerable to damage from environmental stimuli, like ultraviolet (UV) radiation [97]. UV radiation can result in aging, pigmentation, and wrinkle formation [98]. Such abiotic stressors together with some biotic ones, like bacteria, can severely harm skin health. UV radiation is a primary regulator of melanin production, while regulation of melanin production can be utilized for preventing skin-pigmentation, freckling, and age-spot appearance [99]. Seaweed oligosaccharides have been found to be useful in in vitro studies as potential cosmetics for skin-whitening [100,101,102]. They have been found to expressively suppress melanin production [101]. Moreover, such oligosaccharides have been successfully tested in vitro, suggesting their potential use as skin moisturizers [100,101].

A lot of studies have referred to AOS and COS as ‘generally recognized as safe (GRAS)’ [8]. Compared to carrageenan, the COS is more absorbable and less toxic [103]. COS showed no influence on the proliferation of normal ECV304 cells and no direct toxicity to ECV304 cells [55].

## 8. Future Perspective

Oligosaccharides have generated a lot of interest from the food industry due to the important roles they play in human health. Red seaweed is abundant in nature, which deserves to be explored and used due to its high yield and numerous reported biologically active compounds, such as agar and carrageenan. Agar and carrageenan are both good sources of AOS and COS, which are valuable compounds with respect to biological activities. Although much research has been done on oligosaccharides, some loopholes still exist in the current information.

A large number of reported works concentrate on the production of fructo-oligosaccharides and xylo-oligosaccharides using chemical hydrolysis and enzymatic hydrolysis. These products are commercially available, but production and purification of red seaweed oligosaccharides have only been investigated in the laboratory to date. Further evaluation is needed regarding the potential processing of AOS and COS on an industrial scale. Enzymatic production is the better choice in terms of sustainability and long term environmental safety. Furthermore, several chromatographic methods have been adapted to analyze red seaweed oligosaccharides, but no reports about AOS and COS quality control or their chromatographic profile can be found in the available literature at present. Hence, developing a simple and reliable method for the quality control of AOS and COS from red seaweed is direly needed, which would be significant for the application of AOS and COS in improving the quality and performance of functional foods and pharmaceutical products.

The reported physiological benefits of red seaweed oligosaccharides show promise for their potential use in functional foods and pharmaceutical products. The physiological effects of red seaweed oligosaccharides may be attributed to their chemical and physical properties, such as monosaccharide composition, linkage type, glycosidic bond linkages, sulfation, and molecular weight. These correlations and the potential activities of these oligosaccharides have not yet been thoroughly explored. Therefore, fully defining the structure of AOS and COS is of utmost importance in revealing their structure-bioactivity relationships. The chemico-physical properties, biological activities, and molecular mechanisms of action for oligosaccharides derived from red seaweed also require further investigation.

## Supplementary Materials

Supplementary File 1
