# Supplementary material for: Oligosaccharides Derived from Red Seaweed: Production, Properties, and Potential Health and Cosmetic Applications
Source: Molecules. 2018 Sep 25;23(10):2451. doi: 10.3390/molecules23102451 (PMC6222765; doi:10.3390/molecules23102451)
Supplement: Supplementary File 1 [file molecules-23-02451-s001.pdf]

Copyright Clearance Center RightsLink®

Home | Account Info | Help

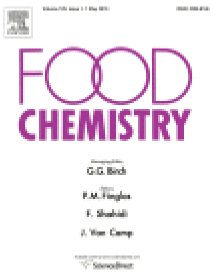

Title: Preparation of bioactive neoagaroligosaccharides through hydrolysis of Gracilaria lemaneiformis agar: A comparative study

Author: Xin-Qi Xu,Bing-Mei Su,Jin-Sheng Xie,Ren-Kuan Li,Jie Yang,Juan Lin,Xiu-Yun Ye

Publication: Food Chemistry

Publisher: Elsevier

Date: 1 February 2018

© 2017 Elsevier Ltd. All rights reserved.

Logged in as:

Kit Leong Cheong
Shantou University

LOGOUT

Custom Permission Request

Review the details below and click 'Submit Request'.
Elsevier will review and respond within 15 business days.

Licensed Content Publisher	Elsevier
Licensed Content Publication	Food Chemistry
Licensed Content Title	Preparation of bioactive neoagaroligosaccharides through hydrolysis of Gracilaria lemaneiformis agar: A comparative study
Licensed Content Author	Xin-Qi Xu,Bing-Mei Su,Jin-Sheng Xie,Ren-Kuan Li,Jie Yang,Juan Lin,Xiu-Yun Ye
Licensed Content Date	1 February 2018
Licensed Content Volume	240
Licensed Content Issue	n/a
Licensed Content Pages	8
Type of Use	reuse in a journal/magazine
Requestor type	medical educational
Portion	figures/tables/illustrations
Number of figures/tables/illustrations	1
Circulation	1
Format	electronic
Are you the author of this Elsevier article?	Yes
Will you be translating?	No
Original figure numbers	Figure 3
Title of the article	Oligosaccharides Derived from Red Seaweed: Production, Properties, and Potential Health and Cosmetic Applications
Publication new article is in	Molecules
Publisher of the new article	MDPI
Author of new article	Kit-Leong Cheong, Hua-Mai Qiu, Hong Du, Yang Liu and Bilal Muhammad Khan
Expected publication date	Sep 2018
Estimated size of new article (number of pages)	18

AMERICAN SOCIETY FOR MICROBIOLOGY

Title: A Novel Agarolytic β-Galactosidase Acts on Agarooligosaccharides for Complete Hydrolysis of Agarose into Monomers

Author: Chan Hyoung Lee,Hee Taek Kim,Eun Ju Yun,Ah Reum Lee,Sa Rang Kim,Jae-Han Kim,In-Geol Choi,Kyoung Heon Kim

Publication: Applied and Environmental Microbiology

Publisher: American Society for Microbiology

Date: Sep 9, 2014

Copyright © 2014, American Society for Microbiology

Logged in as:
Kit Leong Cheong
Shantou University
LOGOUT

Order Completed

Thank you for your order.

This Agreement between Shantou University -- Kit Leong Cheong ("You") and American Society for Microbiology ("American Society for Microbiology") consists of your license details and the terms and conditions provided by American Society for Microbiology and Copyright Clearance Center.

Your confirmation email will contain your order number for future reference.

printable details

License Number	4434131305171
License date	Sep 22, 2018
Licensed Content Publisher	American Society for Microbiology
Licensed Content Publication	Applied and Environmental Microbiology
Licensed Content Title	A Novel Agarolytic β-Galactosidase Acts on Agarooligosaccharides for Complete Hydrolysis of Agarose into Monomers
Licensed Content Author	Chan Hyoung Lee,Hee Taek Kim,Eun Ju Yun,Ah Reum Lee,Sa Rang Kim,Jae-Han Kim,In-Geol Choi,Kyoung Heon Kim
Licensed Content Date	Sep 9, 2014
Licensed Content Volume	80
Licensed Content Issue	19
Licensed Content Pages	9
Type of Use	Journal
Requestor type	Non-profit
Format	Electronic
Portion	Figures/tables/images
Number of figures/tables	1
Will you be translating?	No

Author of this ASM article	No
Order reference number	
Title of new article	Oligosaccharides Derived from Red Seaweed: Production, Properties, and Potential Health and Cosmetic Applications
Publication the new article is in	Molecules
Publisher of new article	MDPI
Author of new article	Kit-Leong Cheong, Hua-Mai Qiu, Hong Du, Yang Liu and Bilal Muhammad Khan
Expected publication date of new article	Sep 2018
Estimated size of new article (pages)	18
Requestor Location	Shantou University<br>Guangdong Province, Shantou City<br><br><br>Shantou, Guangdong 515063<br>China<br>Attn: Shantou University
Publisher Tax ID	38-1616141
Billing Type	Invoice
Billing address	Shantou University<br>243 Da Xue Road,<br>Shantou, Guangdong 515063<br>P.R. China<br>Shantou, China 515063<br>Attn: Shantou University
Total	5.00 USD

ORDER MORE CLOSE WINDOW

Copyright © 2018 Copyright Clearance Center, Inc. All Rights Reserved. Privacy statement. Terms and Conditions.
Comments? We would like to hear from you. E-mail us at customercare@copyright.com

Requestor Location	Shantou University<br>Guangdong Province, Shantou City<br><br><br>Shantou, Guangdong 515063<br>China<br>Attn: Shantou University
Publisher Tax ID	GB 494 6272 12
Total	Not Available

Edit Order Details

Edit Requestor Location   This location may be used to determine your tax liability.

BACK   SUBMIT REQUEST

Copyright © 2018 Copyright Clearance Center, Inc. All Rights Reserved. Privacy statement. Terms and Conditions.
Comments? We would like to hear from you. E-mail us at customercare@copyright.com

Copyright Clearance Center RightsLink®

Home | Account Info | Help

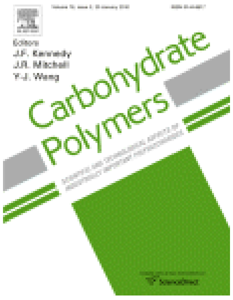

Title: Carrageenans: Biological properties, chemical modifications and structural analysis – A review

Author: Vanessa Leiria Campo,Daniel Fábio Kawano,Dílson Braz da Silva,Ivone Carvalho

Publication: Carbohydrate Polymers

Publisher: Elsevier

Date: 10 June 2009

Copyright © 2009 Elsevier Ltd. All rights reserved.

Logged in as:
Kit Leong Cheong
Shantou University

LOGOUT

Custom Permission Request

Review the details below and click 'Submit Request'.
Elsevier will review and respond within 15 business days.

Licensed Content Publisher	Elsevier
Licensed Content Publication	Carbohydrate Polymers
Licensed Content Title	Carrageenans: Biological properties, chemical modifications and structural analysis – A review
Licensed Content Author	Vanessa Leiria Campo,Daniel Fábio Kawano,Dílson Braz da Silva,Ivone Carvalho
Licensed Content Date	10 June 2009
Licensed Content Volume	77
Licensed Content Issue	2
Licensed Content Pages	14
Type of Use	reuse in a journal/magazine
Requestor type	medical educational
Portion	figures/tables/illustrations
Number of figures/tables/illustrations	3
Circulation	1
Format	electronic
Are you the author of this Elsevier article?	Yes
Will you be translating?	No
Original figure numbers	figure 12, figure 13, and figure 14
Title of the article	Oligosaccharides Derived from Red Seaweed: Production, Properties, and Potential Health and Cosmetic Applications
Publication new article is in	Molecules
Publisher of the new article	MDPI
Author of new article	Kit-Leong Cheong, Hua-Mai Qiu, Hong Du, Yang Liu and Bilal Muhammad Khan
Expected publication date	Sep 2018
Estimated size of new article (number of pages)	18

Requestor Location	Shantou University<br>Guangdong Province, Shantou City<br><br><br>Shantou, Guangdong 515063<br>China<br>Attn: Shantou University
Publisher Tax ID	GB 494 6272 12
Total	Not Available

Edit Order Details

Edit Requestor Location   This location may be used to determine your tax liability.

BACK   SUBMIT REQUEST

Copyright © 2018 Copyright Clearance Center, Inc. All Rights Reserved. Privacy statement. Terms and Conditions.
Comments? We would like to hear from you. E-mail us at customercare@copyright.com
